# Construction of a Covalent Crosslinked Membrane Exhibiting Superhydrophilicity and Underwater Superoleophobicity for the Efficient Separation of High-Viscosity Oil–Water Emulsion Under Gravity

**DOI:** 10.3390/molecules30081840

**Published:** 2025-04-19

**Authors:** Mengxi Zhou, Peiqing Yuan, Xinru Xu, Jingyi Yang

**Affiliations:** 1 International Joint Research Center of Green Energy Chemical Engineering, East China University of Science and Technology, Meilong Road 130, Shanghai 200237, China; y30220228@mail.ecust.edu.cn (M.Z.); xrxu86@ecust.edu.cn (X.X.); 2 State Key Laboratory of Chemical Engineering, East China University of Science and Technology, Meilong Road 130, Shanghai 200237, China; pqyuan@ecust.edu.cn

**Keywords:** superwetting membrane, underwater superoleophobic, emulsion separation, crosslinking, molecular dynamics simulation

## Abstract

The separation of high-viscosity oil–water emulsions remains a global challenge due to ultra-stable interfaces and severe membrane fouling. In this paper, SiO_2_ micro–nanoparticles coated with polyethyleneimine (PEI) were initially loaded onto a stainless steel substrate. This dual-functional design simultaneously modifies surface roughness and wettability. Furthermore, a covalent crosslinking network was created through the Schiff base reaction between PEI and glutaraldehyde (GA) to enhance the stability of the membrane. The membrane exhibits extreme wettability, superhydrophilicity (WCA = 0°), and underwater superoleophobicity (UWOCA = 156.9°), enabling a gravity-driven separation of pump oil emulsions with 99.9% efficiency and a flux of 1006 L·m^−2^·h^−1^. Moreover, molecular dynamics (MD) simulations demonstrate that the SiO_2_-PEI-GA-modified membrane promotes the formation of a stable hydration layer, reduces the oil–layer interaction energy by 85.54%, and exhibits superior underwater oleophobicity compared to the unmodified SSM. Efficiency is maintained at 99.8% after 10 cycles. This study provides a scalable strategy that combines covalent crosslinking with hydrophilic particle modification, effectively addressing the trade-off between separation performance and membrane longevity in the treatment of viscous emulsions.

## 1. Introduction

In the context of accelerated industrial development, oil spills and the discharge of oil-containing wastewater threaten ecosystem balance and pose risks to human health [1,2,3,4]. Although conventional oil–water separation technologies, such as gravity separation, centrifugal separation, biological treatment, floating, adsorption, coagulation, and flocculation have been widely applied [5,6,7,8,9,10], these methods still face challenges in achieving efficient separation for emulsified systems due to the high dispersion and stability of emulsified oil droplets [11].

Superwetting membranes have received considerable attention in the field of oil–water separation due to the high selectivity and low energy consumption advantages [12,13,14]. Among these, superoleophilic and superhydrophobic membranes are prone to oil-phase blockage, leading to membrane fouling [8,15,16,17]. In contrast, superhydrophilic and underwater superoleophobic membranes exhibit better antifouling performance and higher cyclic stability [18,19,20,21,22,23].

Nevertheless, current technologies for superhydrophilic and underwater superoleophobic membranes are still limited by research bottlenecks that hinder their application in large-scale oil–water separation engineering. For instance, the superhydrophilic and underwater superoleophobic membrane developed by Xu et al. achieved a separation efficiency exceeding 99.8% and a flux greater than 1000 L·m^−2^·h^−1^ for immiscible oil–water mixtures. Nevertheless, when processing oil–water emulsions, the separation efficiency remained over 99.6%, while the flux significantly dropped to 53–79 L·m^−2^·h^−1^ [24]. A similar issue was reported by Xiao et al. where the modified membrane demonstrated a separation efficiency above 98% for five oil–water mixtures, with fluxes exceeding 1000 L·m^−2^·h^−1^. Yet, when separating emulsions, stable and small emulsified oil droplets affected the separation efficiency, limiting it to around 93%, with fluxes below 460 L·m^−2^·h^−1^ [25]. These observations highlight that the performance of superhydrophilic and underwater superoleophobic membranes is relatively low when processing oil–water emulsions, with challenges in achieving synergistic optimization between separation efficiency and flux. Furthermore, several studies on superhydrophilic and underwater superoleophobic membranes primarily focus on the separation performance for immiscible oil–water mixtures, lacking in-depth investigations into oil–water emulsions [26,27]. Moreover, meeting high flux demands often relies on vacuum-assisted systems, which increase energy consumption and reduce economic viability. Achieving high separation flux for oil–water emulsions is challenging under standard gravity separation conditions [28,29,30]. Liu et al. reported approximately 350 L·m^−2^·h^−1^ of separation flux when processing oil–water emulsions at an operating pressure of 0.08 MPa [31]. This indicates that even in vacuum conditions, some studies struggle to achieve high separation flux. Pan et al. reported that when using a modified membrane to separate four types of oil-in-water emulsions, the permeation flux was below 160 L m^−2^ h^−1^ bar^−1^. After seven cycles, the separation efficiency of the membrane for kerosene emulsions decreased from 99.7% to 96.9% [8]. Gao et al. employed a composite membrane for separating oil-in-water emulsions, but the separation flux decreased below 70% as the operation progressed [32]. Khan et al. used a modified PVDF membrane for 10 min of continuous oil–water emulsion separation, during which the flux rapidly dropped by 35.55% due to membrane surface fouling [33]. This points out that in some existing studies, long-term cycling of membranes can lead to the degradation of surface wettability, significantly impacting separation performance [8,33,34]. These shortcomings are primarily attributed to the limited antifouling capability, insufficient durability of the membrane materials, and inadequate design of pore structures, which restrict the scalability of the technology in practical industrial applications.

To address these limitations, this study optimized the preparation of oil–water emulsion separation membranes by synergistically regulating surface micro-roughness and chemical modification [34,35]. A stainless steel mesh (SSM) with high mechanical strength was selected as the substrate material. SiO_2_ coated with polyethyleneimine (PEI) was used to create a specific rough structure while providing excellent oleophobic performance. Additionally, glutaraldehyde (GA) was employed to enhance the grafting strength of the modified layer through chemical crosslinking, resulting in a superhydrophilic and underwater superoleophobic membrane that achieves high performance and durability under ambient pressure. The membrane’s antifouling capability, broad applicability, and reusability were demonstrated through tests of wettability, separation efficiency across various oil–water emulsion systems, and cycling experiments. Furthermore, molecular dynamics (MD) simulations were conducted to investigate the influence of the modified layer on the motion trajectories of oil molecules in oil-in-water emulsions. By calculating the interaction energy between oil and the interface, the mechanisms behind the enhanced oleophobic performance were elucidated, providing a theoretical foundation for future research.

## 2. Results and Discussion

### 2.1. Characterization

**Figure 1:**
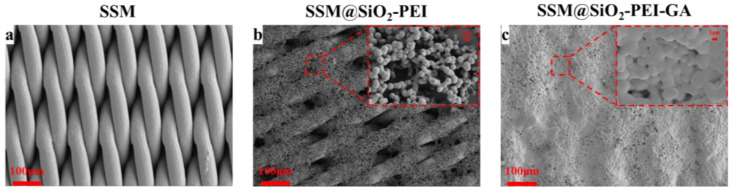
SEM images: (**a**) SSM; (**b**) SSM@SiO_2_-PEI; (**c**) SSM@SiO_2_-PEI-GA.

This study utilized a scanning electron microscope (SEM) to analyze the effects of SiO_2_-PEI and GA on the surface morphology of SSM. The SSM used as the substrate exhibited a mesh structure and a smooth surface (Figure 1a). The surface of SSM@SiO_2_-PEI was coated with a layer of spherical micro–nanoparticles, leading to a reduction in pore diameter and a significant increase in roughness, while large pores still existed (Figure 1b). This indicates that the modified particles have successfully covered the membrane surface. The particle size of the SiO_2_-PEI organic–inorganic hybrid particles was primarily concentrated between 1 and 2 μm. After crosslinking with GA, it was observed that the SSM@SiO_2_-PEI-GA exhibited a smoother surface compared with SSM@SiO_2_-PEI and showed a finer micropore structure and a more compact surface morphology (Figure 1c). The porosity of the SSM, SSM@SiO_2_-PEI, and SSM@SiO_2_-PEI-GA decreased from 38.20% to 34.54% and then to 33.07%. And median pore diameters of the SSM, SSM@SiO_2_-PEI, and SSM@SiO_2_-PEI-GA are 45.5, 32.2, and 36.2 µm, respectively.

**Figure 2:**
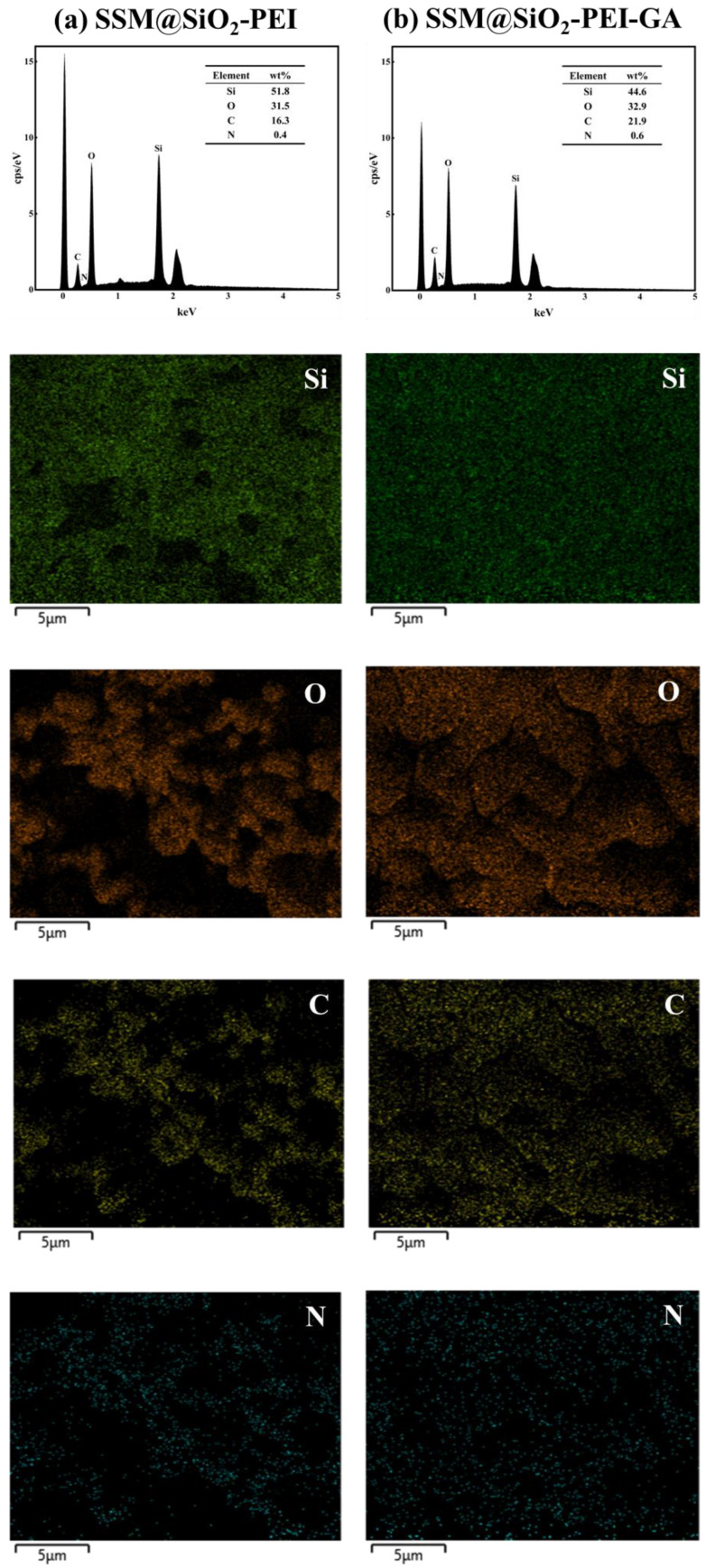
The EDS results and mapping: (**a**) SSM@SiO_2_-PEI; (**b**) SSM@SiO_2_-PEI-GA.

The elemental composition and distribution of the modified membranes were further characterized using energy-dispersive spectroscopy (EDS), as shown in Figure 2. The EDS spectra of SSM@SiO_2_-PEI (Figure 2a) and SSM@SiO_2_-PEI-GA (Figure 2b) revealed the mass percentages of different elements (Si, O, C, and N). For comparison, the EDS spectra of SSM are presented in Figure S1 (Supplementary Materials). After GA modification, the Si content decreased from 51.8 wt% to 44.6 wt%, while the O content increased from 31.5 wt% to 32.9 wt%. This change is attributed to the presence of GA containing aldehyde groups on the surface of the SiO_2_-PEI particles.

**Figure 3:**
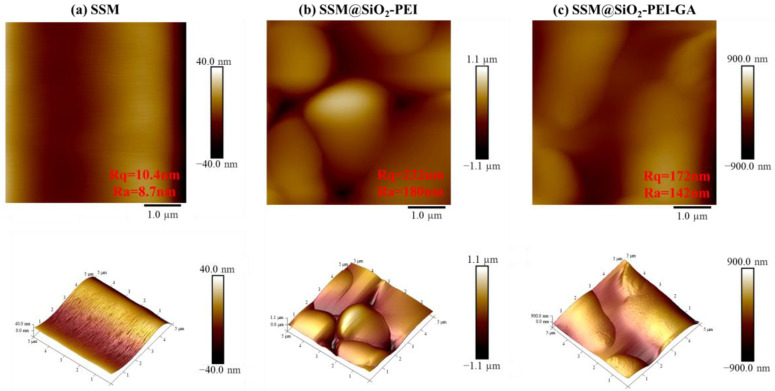
AFM images (2D and 3D): (**a**) SSM; (**b**) SSM@SiO_2_-PEI; (**c**) SSM@SiO_2_-PEI-GA.

To evaluate the roughness and three-dimensional morphology of the membrane surface before and after two modifications, an atomic force microscope (AFM) was utilized to measure and analyze the arithmetic mean roughness (Ra) and root mean square roughness (Rq). The AFM images revealed that the roughness of the original SSM is the smallest, with Rq and Ra values of 10.4 nm and 8.7 nm, respectively (Figure 3a). After crosslinking with GA, the Rq decreased from 232 nm to 172 nm, while the Ra reduced from 180 nm to 142 nm (Figure 3b,c). In agreement with the SEM findings, the SiO_2_-PEI particles initially formed a dense coverage on the surface of the SSM, resulting in an elevated surface roughness. While the overall roughness diminished after crosslinking with GA. This can be attributed to the crosslinker filling the gaps between the particles, leading to a smoother and defect-free surface.

**Figure 4:**
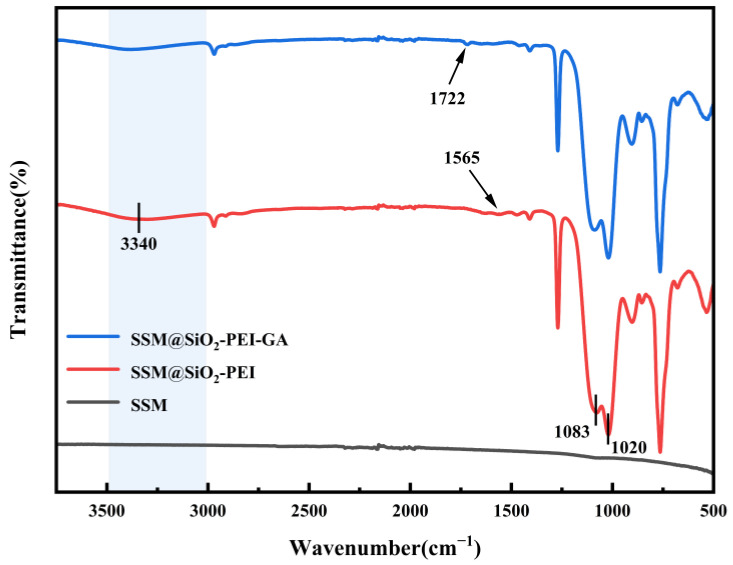
FTIR spectra of SSM, SSM@SiO_2_-PEI, and SSM@SiO_2_-PEI-GA.

The chemical structure of SSM@SiO_2_-PEI and SSM@SiO_2_-PEI-GA was identified using Fourier-transform infrared spectroscopy (FT-IR); the FT-IR spectra are shown in Figure 4. Peaks at 1083 cm^−1^ and 1020 cm^−1^ were caused by the stretching vibrations of Si-O-Si bonds [36], indicating that TMOM underwent hydrolytic condensation to form SiO_2_. The band at 1565 cm^−1^ is attributed to the N-H bending vibrations of primary amines in PEI [37]; this peak was relatively weak due to the low concentration of PEI in the sample. The broadband observed near 3340 cm^−1^ resulted from the overlapping stretching vibrations of N–H and O–H bonds [31,37,38,39]. In the spectrum of SSM@SiO_2_-PEI-GA, a characteristic peak at 1722 cm^−1^ appeared, corresponding to the stretching vibrations of C=O groups in GA [31,40,41]. Additionally, the N-H bending vibration peak of primary amines weakened after grafting GA, further confirming the reaction between primary amines and aldehyde groups. The FT-IR results indicate that SiO_2_-PEI and GA have been successfully grafted onto the SSM.

**Figure 5:**
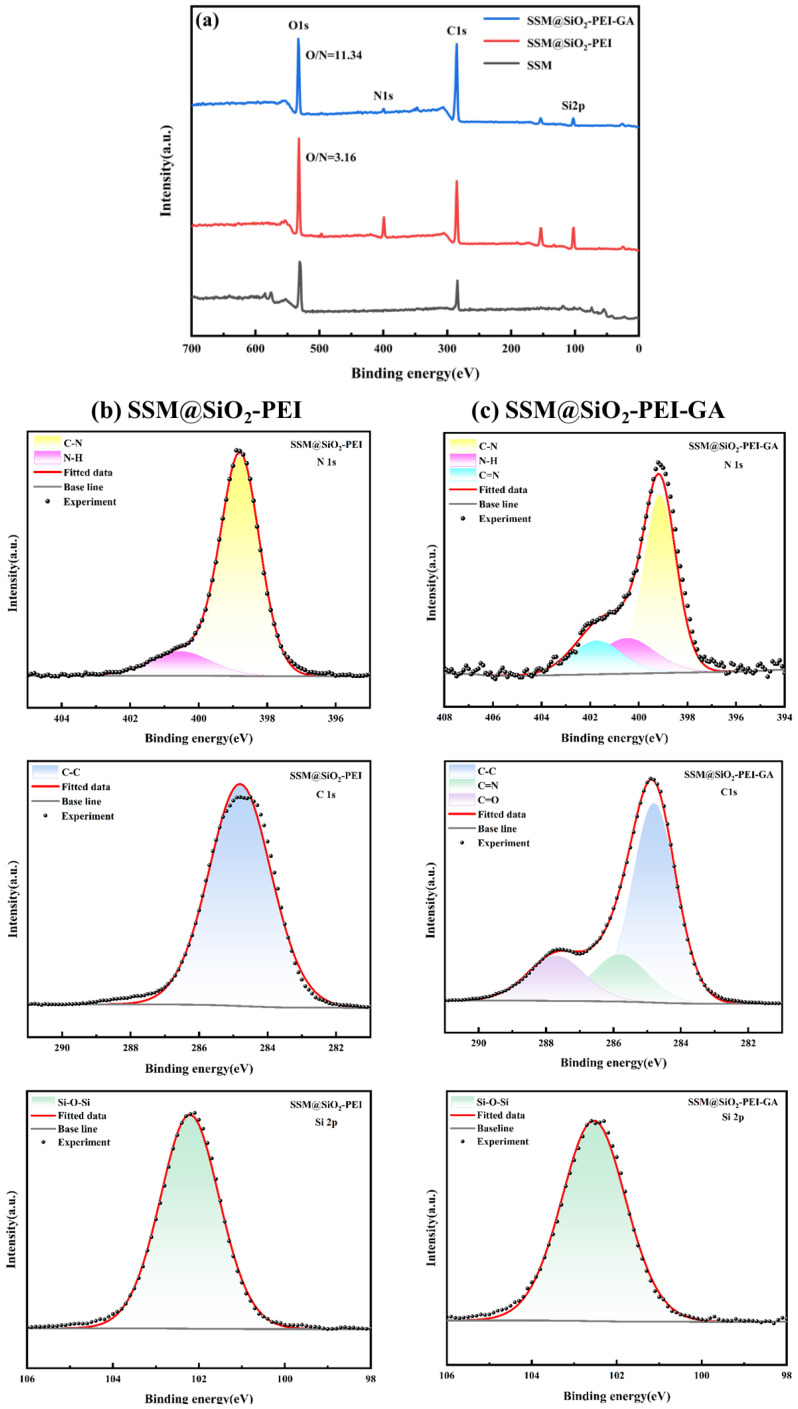
(**a**) XPS survey spectra of SSM, SSM@SiO_2_-PEI, and SSM@SiO_2_-PEI-GA; (**b**) high-resolution XPS N 1s, C 1s, and Si 2p spectra of SSM@SiO_2_-PEI; and (**c**) high-resolution XPS N 1s, C 1s, and Si 2p spectra of SSM@SiO_2_-PEI-GA.

X-ray photoelectron spectroscopy (XPS) was used to further analyze the surface chemical composition, as shown in Figure 5. The spectra indicated the presence of C, N, O, and Si elements in both modified SSM samples (Figure 5a). A detailed analysis of the high-resolution XPS spectra was conducted (Figure 5b,c). In the N1s spectrum of the SSM@SiO_2_-PEI sample, the characteristic peaks at 398.79 eV and 400.55 eV corresponded to C-N and N-H bonds, respectively. For the SSM@SiO_2_-PEI-GA sample, the peaks at 399.14 eV and 400.49 eV in the N1s spectrum were attributed to C-N and N-H bonds [31], confirming that PEI was grafted onto the surface. After grafting GA, the N-H content decreased from 1.36% to 0.47%, and a new peak at 401.73 eV corresponded to the C=N bond [31]. The decrease in N-H content and the formation of the C=N bond confirm the occurrence of the Schiff base reaction between the aldehyde groups of GA and the primary amines of PEI. The C1s spectrum of SSM@SiO_2_-PEI-GA displayed three peaks at 284.80 eV, 285.80 eV, and 287.71 eV, corresponding to C-C, C=N, and C=O bonds, respectively [42]. The presence of the newly formed C=N and C=O bonds, compared to SSM@SiO_2_-PEI, further supports the occurrence of the crosslinking reaction. This reaction can also be corroborated by the weakening of the peak at 1565 cm^−1^ belonging to the N-H bond and the emergence of the peak at 1722 cm^−1^ corresponding to the C=O bond in the FT-IR spectrum. Since XPS can only detect elements within the top layer (≤10 nm) [43], the coverage of SiO_2_ in the modified layer was enhanced after GA grafting, resulting in a significant decrease in the Si 2p peak area from 15.10% to 5.12%. Due to the presence of oxygen from the aldehyde groups in GA, the test results showed that the O content fluctuated minimally, decreasing by only 3.57%. In contrast, the total N content dropped from 9.02% to 2.2%, leading to a significant increase in the calculated O/N ratio from 3.16 to 11.34.

The characterization results indicate that the amine groups of PEI and the aldehyde groups of GA have undergone a Schiff base condensation reaction, successfully producing SSM@SiO_2_-PEI-GA. The formation of the crosslinking network further filled the large pores of the SSM@SiO_2_-PEI, leading to a gradual reduction in the average pore size after both modification steps.

### 2.2. Surface Wettability and Oil Resistance Performance

**Figure 6:**
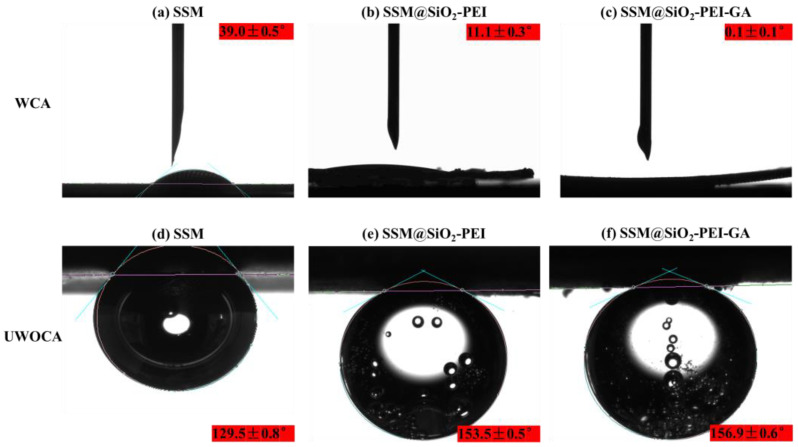
The water contact angles: (**a**) SSM; (**b**) SSM@SiO_2_-PEI; (**c**) SSM@SiO_2_-PEI-GA. The underwater oil contact angles: (**d**) SSM; (**e**) SSM@SiO_2_-PEI; (**f**) SSM@SiO_2_-PEI-GA.

The surface wettability characteristics are crucial for the oil–water separation efficiency of materials. This study assessed the wettability of the membranes by repeated measurements of water contact angle (WCA) and underwater oil contact angle (UWOCA) three times. The WCA of SSM was the highest at 39.0 ± 0.5° (Figure 6a), while the WCA of SSM@SiO_2_-PEI and SSM@SiO_2_-PEI-GA decreased to 11.1 ± 0.3° (Figure 6b) and 0.1 ± 0.1° (Figure 6c), demonstrating superior superhydrophilic performance. When water contacts the modified layer, it could spread, diffuse, and penetrate rapidly, forming a stable hydration layer on the membrane surface. Further investigation into the wettability of the modified membranes was conducted by measuring the UWOCA. Both SSM@SiO_2_-PEI and SSM@SiO_2_-PEI-GA exhibited underwater superoleophobicity with UWOCA values of 153.5 ± 0.5° (Figure 6e) and 156.9 ± 0.6° (Figure 6f), respectively, whereas the UWOCA of the original SSM was only 129.5 ± 0.8° (Figure 6d), confirming the improved underwater oleophobic performance.

### 2.3. Oil–Water Separation Performance

**Figure 7:**
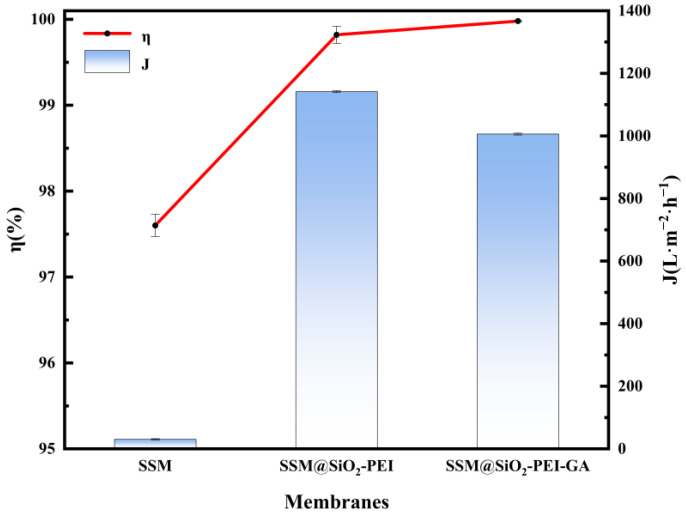
Separation efficiency and flux of SSM, SSM@SiO_2_-PEI, and SSM@SiO_2_-PEI-GA.

In this study, high-viscosity pump oil was selected for the preparation of oil–water emulsion because it contains alkanes, cycloalkanes, aromatic hydrocarbons, olefins, and other compounds, and is not easily volatile, allowing for the creation of complex and stable oil-in-water emulsion. More importantly, this facilitates the investigation of the modified membrane’s applicability in separating high-viscosity oil–water emulsions, which is rare in similar studies. As shown in Figure 7, the original SSM exhibited poor separation performance, with an initial flux of only 30.6 L·m^−2^·h^−1^ (for the first 10 mL). Subsequently, severe membrane fouling occurred, causing the flux to approach zero, and the chemical oxygen demand (COD) of the filtrate reached 21.57 mg·L^−1^. After being modified with both SiO_2_ and PEI, the performance of the SSM@SiO_2_-PEI membrane significantly improved, achieving a separation efficiency of 99.82% and a flux of 1141.64 L·m^−2^·h^−1^. This enhancement is attributed to the SiO_2_ micro–nanoparticles constructing a unique rough structure on the membrane surface [17,44], while the hydrophilic amine groups in PEI further enhance the hydration capability of the surface. The WCA of SSM@SiO_2_-PEI was 11.1 ± 0.3°, and the UWOCA reached 153.5 ± 0.5°, which promoted the formation of a dense hydration layer on the modified membrane [31,45]. This study used a sodium phosphate buffer with multivalent anions to condition PEI, which has a significantly higher ionic strength than monovalent anions, providing multiple sites for electrostatic interactions. This facilitates the connection of phosphates to amine groups, forming polyamine aggregates that serve as favorable nucleation sites for SiO_2_-PEI particles, aiding in the generation of larger and more stable SiO_2_-PEI particles [46]. The combination of special micro–nanostructural features and directional chemical modifications in SSM@SiO_2_-PEI enhances the membrane’s separation capability for oil–water emulsions.

Due to the limited chemical grafting strength, the weak interfacial bonding between SiO_2_-PEI particles and the substrate may lead to easy detachment of the modified layer during dynamic separation. This may adversely affect the long-term operational stability of the membrane material. Crosslinking techniques can prevent the swelling of modified layer particles and enhance the grafting strength of the modified layer [33,45,47,48]. GA, as a crosslinker, can react with amine-containing polymers [49,50], and the abundant amine groups in PEI provide potential reactive sites for crosslinking. The amine groups of PEI and the aldehyde groups of GA will undergo a Schiff base reaction [37,49], which enhances the interfacial bonding strength of the modified material. This improvement strengthens the weak interfacial interactions between the modified layer and the substrate, resulting in a more stable modified structure [50,51]. The SSM@SiO_2_-PEI-GA, reinforced through crosslinking, exhibits optimal overall performance: the COD of the filtrate decreased to 0.22 mg·L^−1^, the separation efficiency increased to 99.9%, and the flux stabilized at 1006.08 L·m^−2^·h^−1^. Additionally, as observed by SEM, the pore size of the SSM@SiO_2_-PEI-GA further decreased, aiding in the blockage of small oil droplets from passing through the pores, thereby enhancing the separation performance for emulsions. The crosslinking technique synergistically optimizes the membrane separation performance by regulating pore size and stabilizing the superwetting surface.

**Figure 8:**
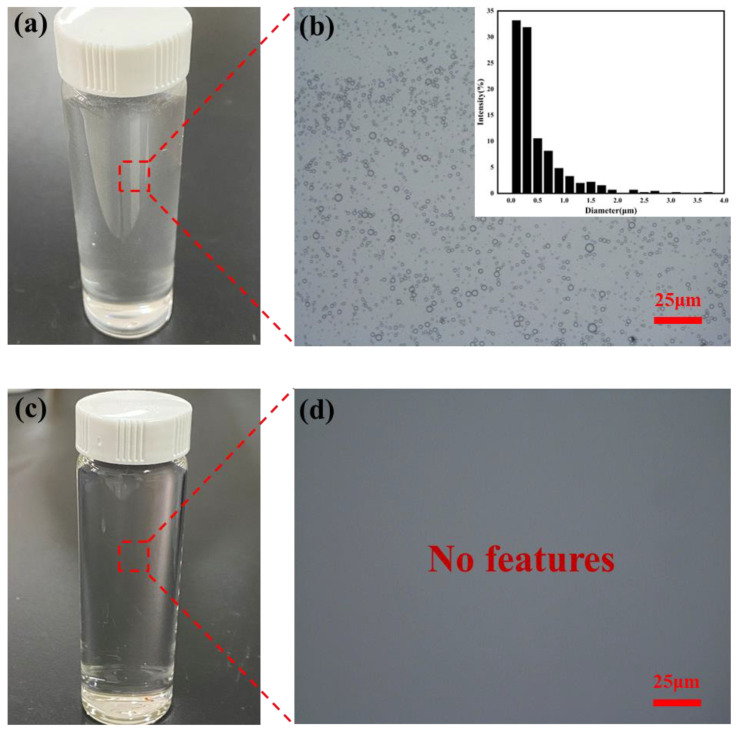
(**a**) Photograph of feed emulsion; (**b**) optical microscopy image and oil droplet size distribution of feed emulsion; (**c**) photograph of filtrate; and (**d**) optical microscopy image of filtrate.

Before separation, the pump oil emulsion appeared milky white (Figure 8a), and widely distributed oil droplets were observed under an optical microscope; the particle size distribution showed that most oil droplets had a diameter of less than 1 µm (Figure 8b). After separation using the SSM@SiO_2_-PEI-GA, the filtrate became transparent (Figure 8c), with almost no detectable oil droplets remaining (Figure 8d). The excellent efficiency of modified membrane in separating pump oil emulsion is further confirmed by this result.

**Figure 9:**
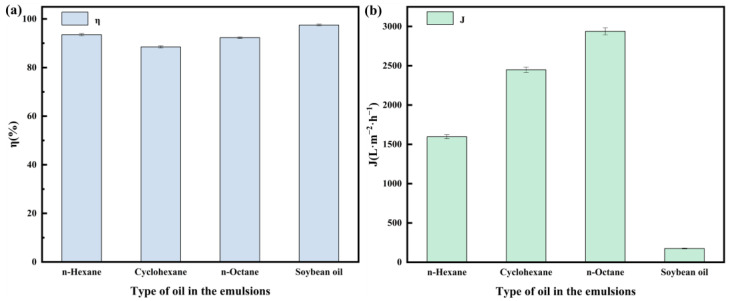
(**a**) Separation efficiency of different oils in the emulsions using SSM@SiO_2_-PEI-GA; (**b**) separation flux of different oils in the emulsions using SSM@SiO_2_-PEI-GA.

To evaluate the separation performance of the SSM@SiO_2_-PEI-GA, four different oil-in-water emulsions were prepared using n-hexane, cyclohexane, n-octane, and soybean oil (Figure 9). The membrane exhibited separation efficiencies of 93.5%, 88.5%, 92.3%, and 97.5% for these emulsions, with corresponding fluxes of 1596.6, 2448.2, 2938.0, and 174.9 L·m^−2^·h^−1^, respectively. Notably, the flux for the soybean oil emulsion was significantly lower than the others. The fouling mechanism of soybean oil on the modified membrane can be attributed to three interrelated factors. First, the triglyceride-dominated composition of soybean oil contains polar functional groups (e.g., ester and carboxylic acid groups) which have a stronger affinity for the amine groups on PEI compared to non-polar mineral oil. This polarity-driven interaction promotes the adsorption of oil droplets onto the modified layer. Secondly, the long-chain triglyceride structure increases the viscosity of the oil, which increases the hydrodynamic resistance and causes oil droplets to accumulate on the membrane surface. This accumulation gradually forms an adherent oil layer that blocks the membrane pores, reducing the effective filtration area and causing a rapid drop in flow. Third, the rough micro–nanostructured surface of the membrane facilitates the physical capture of oil droplets by anchoring polar groups in surface grooves. In addition, the inherent adhesion of soybean oil accelerates irreversible pore blockage, synergistically exacerbating the fouling process [52,53]. These results demonstrate the versatility of the SSM@SiO_2_-PEI-GA in separating a wide range of oil–water emulsions, while highlighting the challenges associated with high-viscosity oils.

### 2.4. Reusability and Antifouling Performance Evaluation

**Figure 10:**
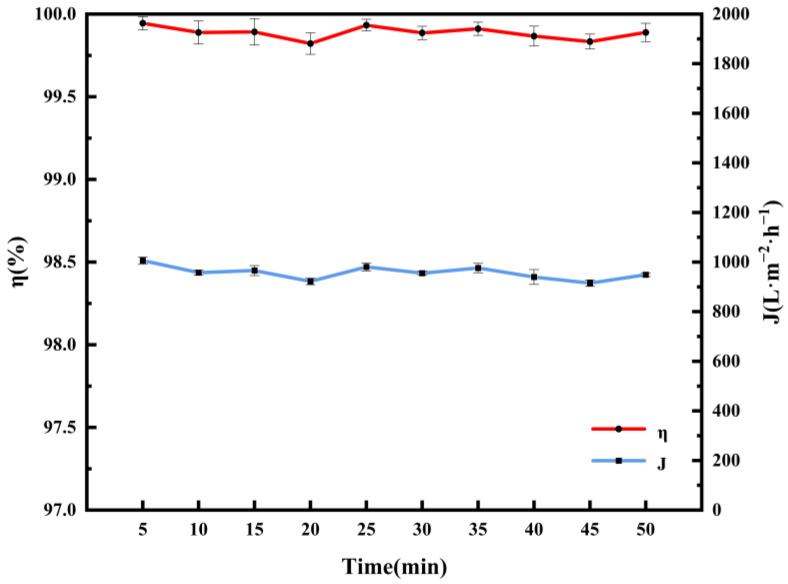
Reusability of SSM@SiO_2_-PEI-GA for pump oil emulsion.

To assess the operational stability of the SSM@SiO_2_-PEI-GA, 10 consecutive separation cycles were performed using a high-viscosity pump oil emulsion (Figure 10). The membrane demonstrated exceptional durability, maintaining over 99.8% separation efficiency throughout all cycles without significant flux loss. Remarkably, surface regeneration was achieved using only 100 mL of ultrapure water for backwashing between cycles, effectively removing residual oil contaminants. This outstanding antifouling performance is due to the robust crosslinked GA-PEI network, which maintains the underwater superoleophobic microstructure of the membrane during repeated use. The combination of structural integrity and ease of regeneration greatly enhances the reusability of the membrane. This feature is a critical advantage for industrial oil–water separation applications, where operational longevity and maintenance costs are paramount.

## 3. Simulation Results and Analysis

**Figure 11:**
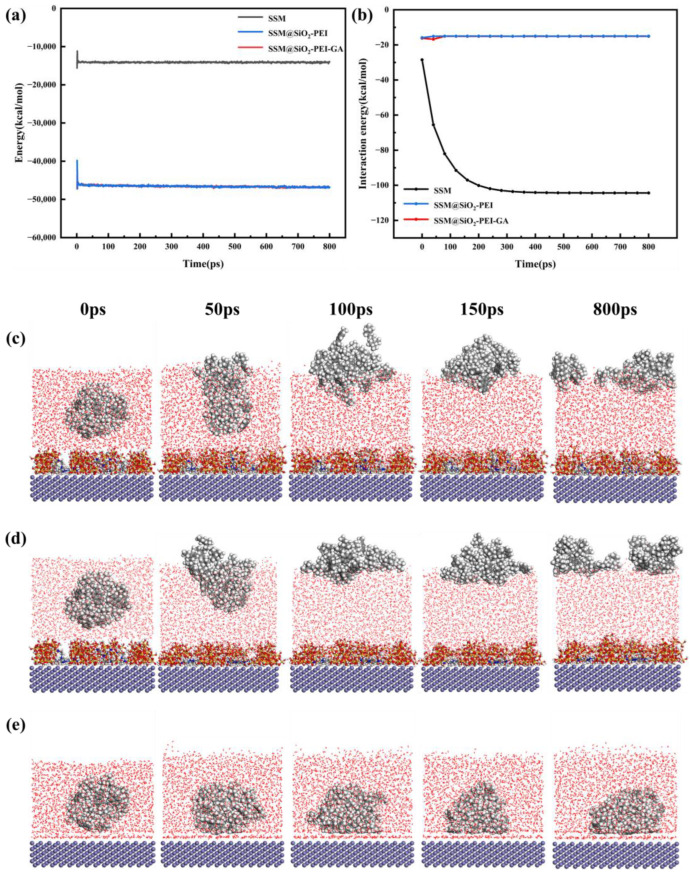
(**a**) The total energy of different models at different times. (**b**) The calculated $E_{Oil/Layer}$ of different models at different times. (**c**) Snapshots of the oil/water-SSM@SiO_2_-PEI-GA model at different times. (**d**) Snapshots of the oil/water-SSM@SiO_2_-PEI model at different times. (**e**) Snapshots of the oil/water-SSM model at different times.

The total energies of the oil/water-SSM, oil/water-SSM@SiO_2_-PEI and oil/water-SSM@SiO_2_-PEI-GA models are shown in Figure 11a, indicating that models reached a stable state before 800 ps. Thus, the dynamic models from 0 to 800 ps were selected for detailed discussion.

As illustrated in Figure 11c,d, water molecules rapidly approached the modified layer in the oil/water-SSM@SiO_2_-PEI and oil/water-SSM@SiO_2_-PEI-GA models; the accumulating water layer forced the oil molecules away from the membrane surface. And according to Figure 11e, the oil molecules in the oil/water-SSM model not only did not show an upward trend but were also closer to the substrate surface; until 800 ps, the oil molecules were still attached to the membrane surface. The comparison of oil residue rates on membrane surfaces indicates that the modified membrane possesses superior hydrophilicity and underwater oleophobic properties.

The interaction energy between the oil molecules and the layer in models was calculated using Equation (1).

(1) $$E_{Oil/Layer}=E_{Oil+Layer}-E_{Oil}-E_{Layer}$$

Here, $E_{Oil/Layer}$ represents the interaction energy between the oil molecules and the layer, $E_{Oil+Layer}$ is the total energy of the oil molecules and the layer, $E_{Oil}$ is the internal energy of the oil molecules, and $E_{Layer}$ is the internal energy of the layer. (For the oil/water-SSM model, *Layer* refers to the SSM. For the oil/water-SSM@SiO_2_-PEI model and oil/water-SSM@SiO_2_-PEI-GA model, *Layer* includes both the SSM and the modified layer.)

The results of MD simulations show that the $E_{Oil/Layer}$ of the oil/water-SSM@SiO_2_-PEI model and the oil/water-SSM@SiO_2_-PEI-GA model are similar, but differ from the oil/water-SSM model (Figure 11b). At the initial time point (0 ps), the $E_{Oil/Layer}$ for the oil/water-SSM model was −28.5 kcal/mol, whereas it was −15.9 kcal/mol for the oil/water-SSM@SiO_2_-PEI model and −16.2 kcal/mol for the oil/water-SSM@SiO_2_-PEI-GA model. After 80 ps, the $E_{Oil/Layer}$ of the oil/water-SSM@SiO_2_-PEI model and the oil/water-SSM@SiO_2_-PEI-GA model changed little, stabilizing around −15 kcal/mol. In contrast, the $E_{Oil/Layer}$ for the oil/water-SSM model sharply increased. And after 320 ps, it tended to stabilize at −104.4 kcal/mol, 5.9 times larger than the oil/water-SSM@SiO_2_-PEI-GA model. The results indicate that the modified layer effectively regulates interfacial wettability by reducing the interaction strength between oil molecules and the membrane surface. It is demonstrated that the modified layer has hydrophilic and underwater oleophobic properties.

**Figure 12:**
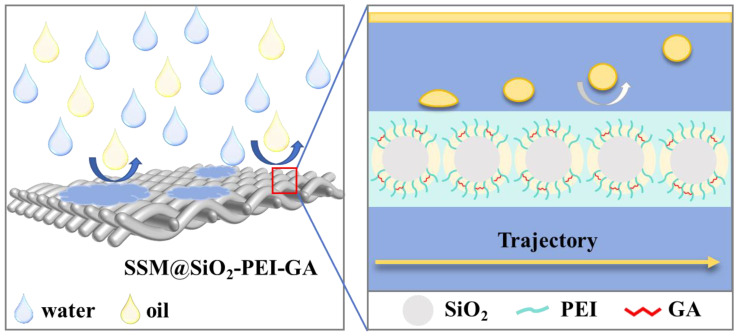
Mechanism of oil–water emulsion separation.

Through MD simulations, the dynamic behavior between oil molecules and the surfaces of the SSM and modified SSM was observed, providing a microscopic perspective to confirm the enhancement of the oleophobic properties of the modified membrane. The pre-hydrated SSM@SiO_2_-PEI-GA rapidly attracts water molecules upon contact with the oil-in-water emulsion, forming a hydration layer. The numerous water molecules adhered to the modified layer occupy the channels where oil droplets would directly contact the membrane surface, preventing oil adhesion to the membrane. Figure 12 illustrates the microscopic process and mechanism behind the underwater oleophobicity of the modified membrane.

## 4. Materials and Methods

### 4.1. Materials

Polyethyleneimine (PEI, M.W. 70,000, 50% in H_2_O), sodium dihydrogen phosphate (NaH_2_PO_4_, AR), glutaraldehyde (GA, AR, 50% in H_2_O), vacuum pump oil (Viscosity Grade 68), and cyclohexane (AR, 99.7%) were purchased from Shanghai Macklin Biochemical Co., Ltd. (Shanghai, China) Trimethoxy(methyl)silane (TMOM, 98%), n-hexane (AR, ≥97%), and n-octane (≥96%) were obtained from Shanghai Aladdin Biochemical Technology Co., Ltd. (Shanghai, China). Disodium hydrogen phosphate (Na_2_HPO_4_, AR, 99%) was sourced from Shanghai Dibo Chemicals Technology Co., Ltd. (Shanghai, China). Hydrochloric acid (HCl, AR, 36.0–38.0%) and acetone (≥99.0%) were acquired from Sinopharm Chemical Reagent Co., Ltd. (Shanghai, China). Ethanol (≥99.7%) was purchased from Shanghai Titan Scientific Co., Ltd. (Shanghai, China). Soybean oil was acquired from Yihai Kerry Arawana Holdings Co., Ltd. (Shanghai, China). The stainless steel mesh was obtained from the market. All aqueous solutions were prepared using ultrapure water.

### 4.2. Preparation of SSM@SiO_2_-PEI-GA

**Figure 13:**
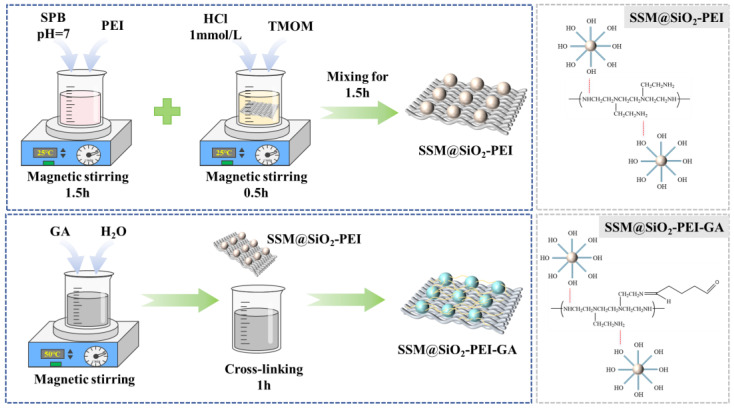
Preparation of SSM@SiO_2_-PEI-GA.

The SSM was cut into circles with a diameter of 5 cm and subjected to ultrasonic cleaning in acetone, ethanol, and ultrapure water for 15 min each. Afterward, the SSM was dried at 60 °C for 8 h to obtain pretreated SSM. Sodium phosphate buffer (SPB) with a pH of 7 was prepared by thoroughly mixing Na_2_HPO_4_ and NaH_2_PO_4_ in water. PEI was added to the SPB and magnetically stirred for 1.5 h to produce a PEI-SPB solution with a PEI concentration of 0.2 wt%. At the same time, TMOM was added to a 1 mmol/L HCl solution to prepare a TMOM-HCl solution with a concentration of 1 mol/L, and the SSM was soaked in this solution for 0.5 h. The PEI-SPB solution and the TMOM-HCl solution were then mixed in a volume ratio of 185:15 and stirred magnetically for 1.5 h. The mixture was allowed to stand at room temperature for 12 h to ensure sufficient loading of the generated SiO_2_-PEI particles onto the SSM membrane. Finally, a 0.2 mol/L GA aqueous solution was prepared and preheated to 50 °C. The SSM@SiO_2_-PEI was immersed in the GA solution at 50 °C for 1 h. The SSM@SiO_2_-PEI-GA was prepared using the aforementioned steps. The detailed preparation process for SSM@SiO_2_-PEI-GA is illustrated in Figure 13.

### 4.3. Oil–Water Separation Experiments

The oil-in-water emulsions were prepared by mixing the oil phase and water at a mass ratio of 1:99, followed by stirring for 20 min in a homogenizer at 2000 r/min. These emulsions were then mixed with water at a mass ratio of 1:25 and stirred for 10 min to obtain the desired oil-in-water emulsions. The oil phase includes pump oil, n-hexane, cyclohexane, n-octane, and soybean oil. The particle size distribution of both the pump oil emulsion and the filtrate was observed using an optical microscope (Nikon Eclipse LV100N POL, Tokyo, Japan).

**Figure 14:**
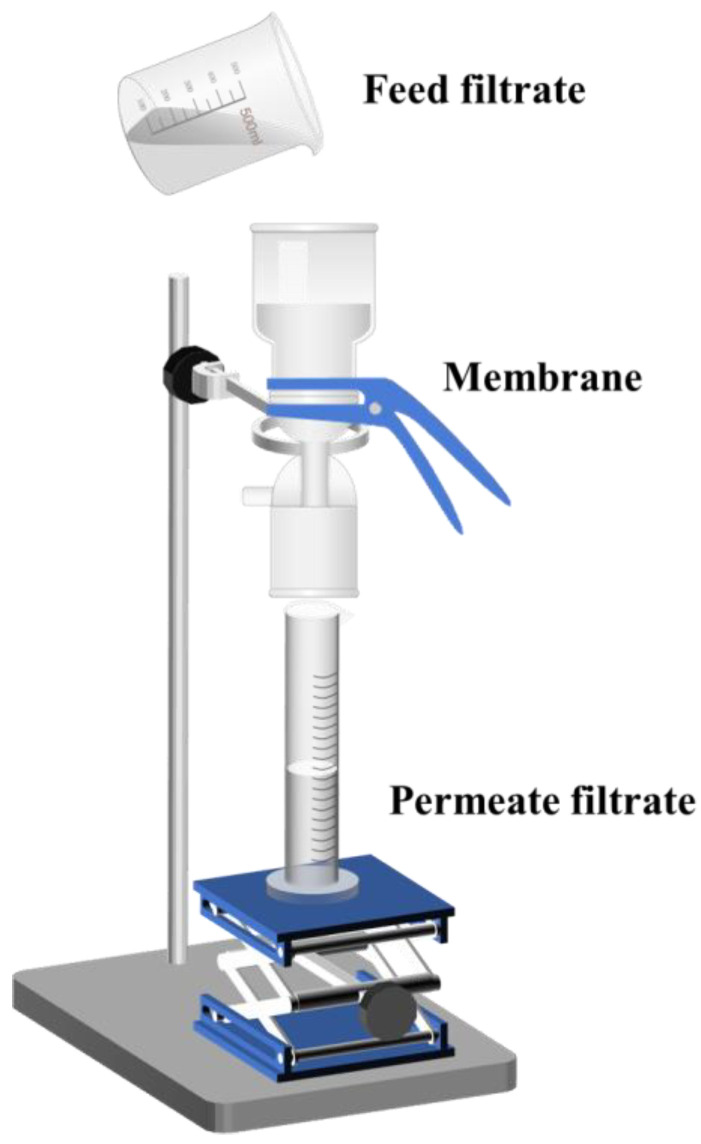
Schematic diagram of the emulsion separated by a membrane.

The tests for the membrane’s separation performance were conducted under gravity, and the separation apparatus is shown in Figure 14. The membrane was installed in a vertical filtration device, and the oil-in-water emulsion was poured into the upper filter cup. After 3.5 min, the filtrate collection commenced, and the filtration time and the COD value for every 100 mL of filtrate were recorded. The COD values discussed in this study were determined using a chemical oxygen demand analyzer (LH-T3COD, Shanghai Lianhua Industrial Co., Ltd., Shanghai, China) based on the potassium dichromate spectrophotometric method. The separation efficiency was calculated using Equation (2).

(2) $$\eta =\left(1-\frac{{COD}_{p}}{{COD}_{f}}\right)\times 100\%$$

Here, η is the separation efficiency (%), and ${COD}_{p}$ and ${COD}_{f}$ are the COD values of the permeate and the feed filtrate (mg/L), respectively.

The separation flux was calculated using Equation (3).

(3) $$J=\frac{V}{S\times \Delta t}$$

Here, J is the flux (L·m^−2^·h^−1^), V is the volume of permeate filtrate (L), S is the effective area of the membrane (m^2^), and ∆t is the permeation time (h).

### 4.4. Characterization of Membranes

This study used a scanning electron microscope (SEM, Zeiss Sigma 500, Jena, Germany) to observe the microstructural morphology of the membrane surface. The porosity was measured by a mercury intrusion porosimeter (MIP, Micromeritics AutoPore IV 9520, Norcross, GE, USA). The elemental composition was analyzed using energy-dispersive spectroscopy (EDS, Oxford Xplore 30, Oxford, UK), while an atomic force microscope (AFM, Bruker Dimension Icon, Karlsruhe, Germany) was utilized to examine the three-dimensional morphology of the membrane surface. The chemical composition of the membrane surface was analyzed using Fourier-transform infrared spectroscopy (FTIR, Thermo Fisher Scientific Nicolet iS20, Waltham, MA, USA) and X-ray photoelectron spectroscopy (XPS, Thermo Fisher Scientific K-Alpha, Waltham, MA, USA). The static water contact angles (WCAs) in air and underwater oil contact angles (UWOCAs) of the membrane were measured using a contact angle goniometer (Dataphysics OCA20, Filderstadt, Germany) to assess the wettability of the modified membrane.

### 4.5. Molecular Dynamics Simulation

This study conducted MD simulations using Materials Studio 6.1. Geometry optimization, dynamic, and energy calculation were accomplished within the Forcite module, and the simulations were performed under the COMPASS III force field. Models for oil/water-SSM, oil/water-SSM@SiO_2_-PEI, and oil/water-SSM@SiO_2_-PEI-GA were established to explore changes in the membrane’s wettability. This was accomplished by observing the motion trajectories of oil molecules and calculating the interaction energies between the oil molecules and the membrane surface.

Initially, a mixed oil-in-water model was created using the Amorphous Cell module, consisting of 39 oil molecules, including C_6_H_14_ (n-hexane), C_7_H_16_ (3-ethylpentane), C_8_H_18_ (3,3-dimethylhexane), C_9_H_20_ (2,4,4-trimethylpentane), C_6_H_12_ (cyclohexane), C_7_H_14_ (cycloheptane), C_9_H_12_ (4-ethyl toluene), and C_11_H_10_ (2-methylnaphthalene). The box was filled with water molecules (2392 H_2_O molecules) using the Packing tool. And the system was relaxed to a steady state under an NPT ensemble at 298 K and 0.1 MPa, yielding an oil-in-water model with reasonable density. The substrate in the model was constructed by cutting the Fe standard structure and using the Super Cell tool. SiO_2_-PEI and SiO_2_-PEI-GA modified layers were constructed from the characterization results and arranged on top of the substrate. The constructed models were then geometrically optimized and annealed to establish the SSM@SiO_2_-PEI model and SSM@SiO_2_-PEI-GA model. The oil-in-water model was then placed above the SSM, SSM@SiO_2_-PEI, and SSM@SiO_2_-PEI-GA models, with a distance of 20 Å from the spherical oil droplets to the Fe substrate and modified layers in the models. The oil/water-SSM, oil/water-SSM@SiO_2_-PEI, and oil/water-SSM@SiO_2_-PEI-GA models were obtained. In this study, the lattice dimensions were set to 49 Å × 48 Å × 100 Å. After geometry optimization, NVT ensemble MD simulations were conducted for 800 ps at 298 K and 0.1 MPa conditions.

## 5. Conclusions

This study presents a synergistic strategy for fabricating high-performance oil–water separation membranes by integrating the interfacial microstructure and chemical crosslinking techniques. A rough surface structure was constructed on an SSM substrate by loading SiO_2_-PEI micro–nanoparticles, which significantly enhanced the surface hydrophilicity. Subsequent crosslinking via Schiff base reactions between GA and PEI enhanced the grafting stability of the modified layer, resulting in a durable oil-in-water emulsion separation membrane (SSM@SiO_2_-PEI-GA). The optimized membrane exhibited exceptional superhydrophilicity (WCA = 0.1 ± 0.1°) and underwater superoleophobicity (UWOCA = 156.9 ± 0.6°), enabling a gravity-driven separation of high-viscosity pump oil emulsion with 99.9% efficiency and a flux exceeding 1000 L·m^−2^·h^−1^. Notably, the membrane maintained 99.8% separation efficiency after 10 operating cycles, demonstrating superior antifouling resistance and reusability. MD simulations were used to elucidate the antifouling mechanism. The results showed that the formation of a robust hydration layer on the modified surface significantly reduced the oil–layer interaction energy compared to the SSM, thus effectively inhibiting oil adhesion. This dual optimization of physical microstructure and chemical stability provides a paradigm for designing separation membranes that simultaneously achieve high efficiency, high flux, and long-term durability. The proposed methodology not only advances the fundamental understanding of oil-resistant interfaces but also demonstrates practical potential for industrial applications, particularly in the tertiary treatment of oil-contaminated wastewater from the petrochemical and marine industries.

## Data Availability

Data are contained within the article or the Supplementary Materials.

## Supplementary Materials

The following supporting information can be downloaded at: https://www.mdpi.com/article/10.3390/molecules30081840/s1, Figure S1. The EDS results and mapping of SSM.
