# Supplementary material for: Construction of a Covalent Crosslinked Membrane Exhibiting Superhydrophilicity and Underwater Superoleophobicity for the Efficient Separation of High-Viscosity Oil–Water Emulsion Under Gravity
Source: Molecules. 2025 Apr 19;30(8):1840. doi: 10.3390/molecules30081840 (PMC12029613; doi:10.3390/molecules30081840)
Supplement: Supplementary file 1 [file molecules-30-01840-s001.zip › molecules-3584709-supplementary.pdf]

Supporting information

Construction of a Covalent Crosslinked Membrane Exhibiting Superhydrophilicity and Underwater Superoleophobicity for the Efficient Separation of High-Viscosity Oil–Water Emulsion Under Gravity

Mengxi Zhou [1], Peiqing Yuan [2], Xinru Xu [1] and Jingyi Yang [1,*]

[1] International Joint Research Center of Green Energy Chemical Engineering, East China University of Science and Technology, Meilong Road 130, Shanghai 200237, China; y30220228@mail.ecust.edu.cn (M.Z.); xrxu86@ecust.edu.cn (X.X.)

[2] State Key Laboratory of Chemical Engineering, East China University of Science and Technology,
Meilong Road 130, Shanghai 200237, China; pqyuan@ecust.edu.cn

* Correspondence: jyyang@ecust.edu.cn

Text S1

Characterization of SSM

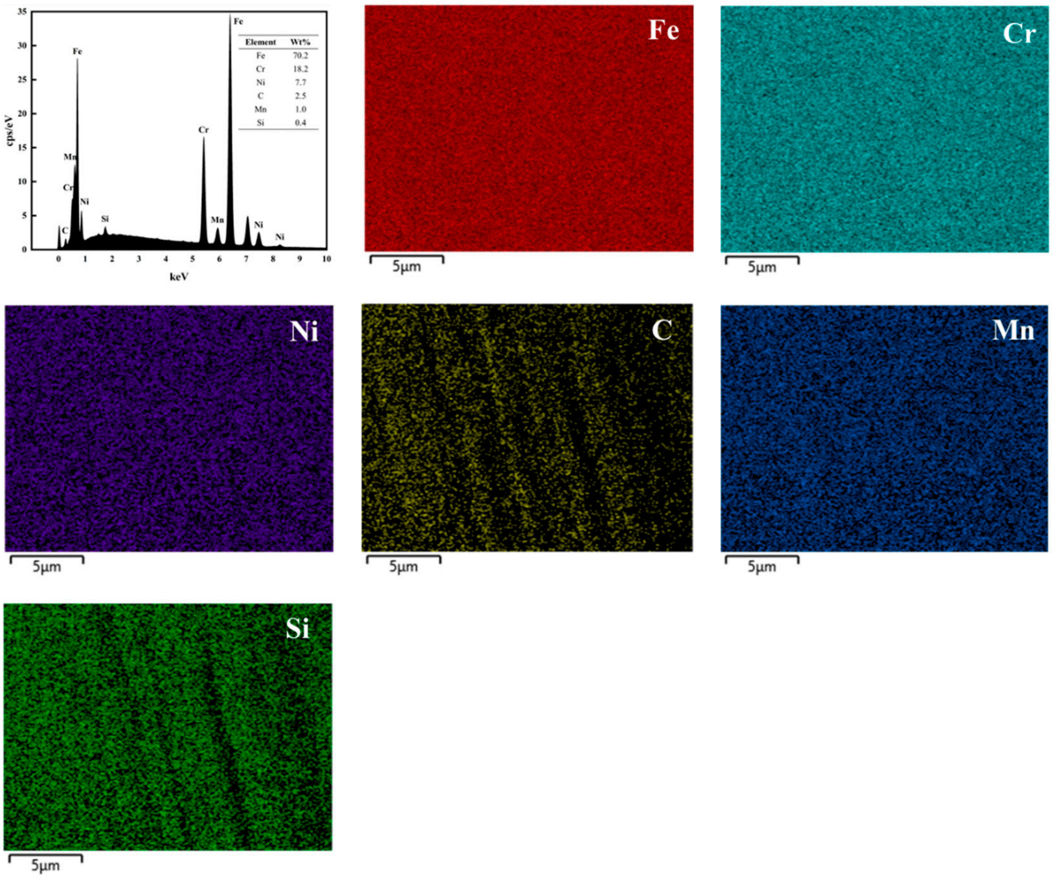

Figure S1. The EDS results and mapping of SSM
